# Calcium Absorption from Food Products: Food Matrix Effects

**DOI:** 10.3390/nu14010180

**Published:** 2021-12-30

**Authors:** Blerina Shkembi, Thom Huppertz

**Affiliations:** 1 Food Quality & Design Group, Wageningen University & Research, 6708 WG Wageningen, The Netherlands; blerina.shkembi@wur.nl; 2 FrieslandCampina, 3818 LE Amersfoort, The Netherlands

**Keywords:** calcium, food, dairy, absorption, gastric, intestinal, complexation

## Abstract

This article reviews physicochemical aspects of calcium absorption from foods. Notable differences are observed between different food products in relation to calcium absorption, which range from <10% to >50% of calcium in the foods. These differences can be related to the interactions of calcium with other food components in the food matrix, which are affected by various factors, including fermentation, and how these are affected by the conditions encountered in the gastrointestinal tract. Calcium absorption in the intestine requires calcium to be in an ionized form. The low pH in the stomach is critical for solubilization and ionization of calcium salts present in foods, although calcium oxalate complexes remain insoluble and thus poorly absorbable. In addition, the rate of gastric transit can strongly affect fractional absorption of calcium and a phased release of calcium into the intestine, resulting in higher absorption levels. Dairy products are the main natural sources of dietary calcium in many diets worldwide, which is attributable to their ability to provide high levels of absorbable calcium in a single serving. For calcium from other food products, lower levels of absorbable calcium can limit contributions to bodily calcium requirements.

## 1. Introduction

The human body requires many nutrients for health and development. While some nutrients can be produced by the body, many cannot and these essential nutrients thus need to be supplied via exogenous sources, which are primarily via the diet. Poor quality diets can lead to deficiencies in several essential nutrients, with calcium being a prominent risk factor for deficiency. The World Health Organization (WHO) suggests a recommended daily intake (RDI) of 1000 mg calcium per day for young adults and 1300 mg/d for men over 65 years, for postmenopausal women and for children aged 9 to 18 years [1]. Such an intake, however, is not met in all parts of the world. In many low- and middle-income countries and territories, a large proportion of the population, particularly in rural areas, does not get enough dietary calcium. Table 1 presents the data on the average daily calcium intake and the main sources of calcium in different countries. In Europe and North America, the daily calcium intake is relatively high, with Greece, the Netherlands and Denmark in first, second and third positions, respectively (1039, 1033 and 1011 mg/day), while China and India (rural and urban areas) represent the territories characterized by the lowest calcium intake (369, 269 and 308 mg/day), far below the aforementioned RDI’s suggested by WHO.

Table 1 also includes the main dietary calcium sources listed for the different countries and regions in order of contribution. From this, it is clear that in all countries and regions with high calcium intake for the adult population, milk and dairy products are the main contributor. This is in line with reports that consumption of milk and other dairy products make a great contribution to calcium intake [2,3,4,5,6,7,8]. For example, milk and derivatives provide about 75% of dietary calcium in the United States and about 58% in the Netherlands [9], while in China only 6.7% of calcium comes from dairy products and the main sources of calcium in this country are vegetables (30.2%), legumes (16.7%) and cereals (14.6%) [10].

Milk and dairy products (e.g., cheese, butter, cream, yoghurt and kefir) are an essential component of the diet of billions of people worldwide [17] and do proportionally contribute a lot of calcium. For instance, 100 g of cheese contains can contain up to 1 g of calcium, 100 g of milk and yoghurt contain between 100 mg and 180 mg, whereas 100 g of cereals usually provide 30 mg of calcium [9]. Some other calcium-rich foods, such as kale, broccoli and watercress contain between 100 and 150 mg of calcium per 100 g [9]. In addition to consumption of food naturally rich in calcium, intake via calcium-fortified foods and beverages or calcium supplements is also an option [18,19]. However, any food product should not be considered as solely a source of a single nutrient; e.g., milk is also a rich source of other essential nutrients, e.g., phosphorous, selenium, potassium, magnesium and zinc and vitamins B1, B2 and B12 [20].

The RDI for calcium is notably higher than daily endogenous losses, which is due to the fact that not all the ingested calcium is absorbed in the gastrointestinal tract; hence, an accurate evaluation of calcium sources should not only be on the basis of calcium content, but also on bioavailability [21]. The bioavailability is the proportion of a nutrient present in a food that is actually absorbed and utilized in different metabolic processes [22]. Calcium bioavailability can be influenced by various physiological factors, e.g., vitamin D status, age, pregnancy, disease, but also by the food’s composition of nutrients [21,23]. In this paper, we review calcium absorption from a food matrix perspective, focusing on interactions of calcium with other components in the food matrix and how these are affected in the gastrointestinal tract. For this purpose, the review starts with a brief overview of the importance of dietary calcium and calcium absorption mechanisms and subsequently discusses calcium absorption from foods in this perspective. Given the large focus in literature on dairy products, this product category is dealt with more extensively, and provides an excellent comparative basis for other foods. In the final chapter, we also consider dietary calcium from the perspective of sustainability, i.e., in terms of the carbon footprint associated with calcium intake from different food sources.

## 2. The Importance of Dietary Calcium for Human Health

Calcium is one of the most studied minerals in relation to human health, and is also the most abundant mineral in the human body. The total calcium content in the body of an individual increases from ~25–30 g at birth to ~1000–1500 g in a well-fed adult. Calcium is an essential nutrient which is not synthesized by the human body, and all the calcium necessary for growth, as well as to compensate for daily losses, must be supplied via exogenous sources, which is mainly via food [24]. More than 99% of calcium in the human body is stored in the bone tissue and teeth in the form of hydroxyapatite, which plays a key structural function by providing rigidity to the skeletal system [21,25]. The remaining (less than 1%) bodily calcium is found in the soft tissues and in bodily fluids, i.e., blood and extracellular fluids. In the soft tissues, calcium is mostly stored in various cytoplasmic organelles. In the blood, calcium is present in three different forms: 45–50% is present as free cations, 40–45% is bound to plasma proteins and 8–10% is dissolved and complexed with other ions, such as citrate and lactate. Serum calcium concentration is usually maintained around 1.0–1.2 mmol/L in healthy subjects and is controlled by the action of parathyroid hormone, vitamin D and calcitonin, which regulate intestinal calcium absorption, renal excretion or reabsorption and removal or incorporation into bone tissue, respectively [26].

In addition to its structural function, calcium is involved in a multitude of vital functions, including fertilization, blood coagulation, muscle contraction, transmission of nerve impulses, secretory activity, cell death, immune response, cell differentiation and enzyme activation [8,21,27]. Attaining and retaining the required amount of calcium is essential for the development, strength and density of bones in children and for the prevention of bone loss and osteoporotic fractures in elderly people [28,29,30]. An adequate calcium intake contributes also to reduce the risk of different chronic diseases, e.g., hypertension, hypercholesterolemia, colon cancer, kidney stones and abdominal obesity [8,31,32,33]. Of course, calcium intake alone is not sufficient to achieve these vital functions, as calcium needs to be absorbed. This is covered in Section 3.

## 3. Absorption of Calcium: Physiological and Physicochemical Perspectives

### 3.1. Physiological Perspectives of Calcium Absorption

As outlined previously, calcium can only play its role in the many vital functions if it is absorbed. Calcium is absorbed in the gastrointestinal tract in the ionized form, i.e., as Ca^2+^ [34,35,36]. Intestinal calcium absorption is an essential process involved in the maintenance of Ca^2+^ homeostasis [37] and occurs through two distinct transport mechanisms: transcellular active saturable transport and paracellular passive non-saturable transport [38]. Transcellular transport occurs in the duodenum and upper jejunum, is stimulated by vitamin D and consists of three essential steps, i.e., (1) calcium entry into the cell (via positive electrochemical gradient), (2) diffusion and (3) excretion from the cell. The entry of calcium into the brush border membrane of enterocytes is mainly mediated by the epithelial TRPV6, located in the apical region [39,40] and high levels of TRPV6 have been detected in the duodenum and colon of humans, rats and mice [40,41,42].

Once inside the cell, calcium is transported to the basolateral membrane bound to the buffer protein calbindin-D9K (CaBP-9K), which has a high affinity for Ca^2+^. Calmodulin, another calcium-binding protein, may contribute to the intracellular translocation of calcium, but to a lesser extent than calbindin-D9K [27]. Calcium is extruded from the epithelial cell to the interstitial space by the action of a calcium-pumping ATPase (PMCA1) and by means of a sodium-calcium exchanger (NCX1), which are located in the basolateral membrane [43,44]. PMCA1 is essential for calcium absorption and in humans is present in the duodenum, ileum and colon [45]. PMCA1 activity is regulated by calmodulin (CaM), calbindin-D28K (CaBP-28K) and calcium [14].

Paracellular passive diffusion of calcium mainly occurs in the small intestine, is not saturable and is vitamin D-independent; absorption via paracellular diffusion increases following increased calcium intake [46,47,48,49] and when calcium intake is high paracellular absorption predominates [50]. Paracellular absorption occurs through tight junctions throughout the small intestine [51,52]. Tight junctions are intercellular structures present in the apical region of enterocytes, which regulate paracellular transport of ions and molecules [53]. In rats, most of the calcium passive absorption occurs in the ileum (65–88%) [54,55]. In addition to these physiological aspects of calcium absorption, there are also various physicochemical factors that play a key role. These are covered in Section 3.2.

### 3.2. Physicochemical Aspects of Calcium Absorption

To understand physicochemical factors governing absorption of calcium from foods, it is important to understand both the locations and time-scale of absorption. The small intestine is responsible for more than 90% of the total calcium uptake in humans, whereas ~3–6% of calcium is absorbed in the colon, depending on calcium load [56]. In the colon, calcium is absorbed by both an active and passive process [57]. When calcium intake is adequate or high, in the small intestine most of the calcium uptake occurs in the ileum [55]. The reason why most of the calcium is absorbed in the ileum is to be found in the fact that the transit time of chyme in this last part of the small intestine is significantly longer than in the duodenum and jejunum. As shown in Table 2, the transit time in humans was ~78 min in the ileum, ~102 min in jejunum and ~15 min the duodenum [58].

Rats have also been used often for calcium absorption studies, but as outlined in Table 2, transit times differ notably from human. Although total transit time in the small intestine does not differ notably between rats and humans (Table 2), the ileal transit time is notably longer and the jejunal transit time is notably shorter in rats. These differences could significantly influence the absorption of calcium in the different tracts of the small intestine between the two species, and thus stress the need for caution when translating data from rat studies to the human situation. The importance of consideration of transit times was shown in an in vitro study simulating a canine digestive process, showing that intestinal calcium absorption was lower when the transit time was faster [59].

Another important factor affecting calcium absorption is the concentration of calcium in a specific intestinal segment. Vergne-Marini et al. [60] showed that the ileal absorption of calcium in humans increases proportionally to the increase in luminal concentration, following a linear trend for solutions of calcium gluconate. These authors also showed that, under physiological conditions, the absorption rate in the ileum was slightly lower than in the jejunum with less concentrated calcium gluconate solutions (1 mM and 5 mM) and slightly higher than in the jejunum with more concentrated calcium gluconate solutions (15 and 20 mM). While the work of Vergne-Marini et al. [60] suggests that calcium absorption correlated linearly with calcium concentration, it should be realized that calcium gluconate solution may be considered as a rather ideal system, with most calcium in ionic form and little or no interference from other components. In the chyme of food products, both physical and chemical interference with calcium absorption can occur.

Before it can be absorbed, calcium must be in solution in the ionized form; while some calcium may already be in ionized form when consumed, solubilization and ionization of calcium from foods often occurs in the acidic environment of the stomach, where solubility of calcium salts and complexes increases [14,34,36]. When the chyme is emptied from the stomach into the duodenum, the pH rapidly changes from highly acidic pH in the stomach (1–3) to about pH 6. The intraluminal pH continues to gradually rise, reaching alkaline levels in the terminal ileum. In the cecum, the pH decreases to ~6.4, after which it gradually increases again along the transit into the colon, reaching pH 7 in the rectum [61]. Because of this change in pH between the gastric and the intestinal part, a reduced amount of calcium may be in ionized form. Bronner and Pansu [57] reported that some calcium may reprecipitate due to the alkaline conditions of the ileum. However, even when calcium is precipitated due to the alkaline environment, some calcium ions remain in solution. These will then be absorbed, and other calcium ions will be released in solution, but this process may be time-consuming. The total calcium uptake therefore depends on a combination of factors: the local solubility, the luminal concentration and the sojourn time in the specific intestinal segment [60,62].

Overall, it is clear that calcium absorption is governed by a number of factors affecting three key aspects, i.e., the concentration of ionized calcium, the rate of absorption of ionized calcium and the transit time of the material through the intestine.

The concentration of ionized calcium is mainly governed by salt equilibria and pH. While calcium chloride, for instance, has high solubility which is virtually independent of pH, dietary calcium is present in food matrices often containing calcium salts of phosphate, carbonate, citrate, phytate or oxalate or complexes of calcium with proteins. These have notably lower solubility, and a strong pH-dependence of solubility, which can lead to supersaturation in the intestine, as will be described in subsequent sections. If ionized calcium is absorbed, via either the trans- or paracellular route, some further calcium salts can dissolve and further calcium can again ionize. Hence, the rate of solubilization and ionization of calcium under these conditions will also play an important role in calcium absorption. Diffusion rates in the chyme are also important for enabling calcium absorption and are influenced notably by product types. In the next Section 4, Section 5, Section 6 and Section 7 absorption of calcium from various sources is covered. Section 4 will cover mechanistic insights attained from absorption from different calcium salts, learnings of which are applied in understanding different food products covered in Section 6.

## 4. Calcium Absorption from Calcium Salts

While for many people calcium intake is exclusively in the form of dietary calcium, there are cases when dietary calcium intake is insufficient, calcium supplements can be a help in the prevention or treatment of calcium deficiency [63]. They are commonly recommended to older adults, particularly for postmenopausal women or amenorrheic women for the prevention of osteoporosis [64,65]. Calcium supplements can be in the form of different calcium salts, and while not the primary aim of this paper, studies on supplements illustrate well how calcium absorption differs between different calcium sources and is thus covered briefly in this section.

Calcium carbonate and calcium citrate are the most widely used calcium forms to the consumers [66]. Some other available calcium supplements include calcium phosphates, calcium gluconate, calcium fumarate, calcium malate, calcium lactate and some mixed salts, such as calcium lactate malate or calcium lactate citrate. These mixed salts have also been used to fortify different beverages because of their good water solubility [67]. Calcium lactate and calcium gluconate are more soluble in water than calcium carbonate and citrate, but they are not considered practical for oral supplements because they contain less elemental calcium [67,68] and many tablets need to be consumed to reach the same dose as calcium carbonate [69]. Calcium carbonate contains the highest amount of elemental calcium (around 40%) compared to calcium citrate and other salts, and is also the less expensive calcium source on the market [70,71]. Data in Table 3 show that the solubility of calcium carbonate, calcium citrate and calcium phosphate increased when pH decreased, and the calculated solubility of calcium depends on the solubility of the salt and the calcium content of the salt itself.

In addition to solubility, absorption is another parameter that must be considered when evaluating the calcium supplement effectiveness. Heaney et al. [73] reported that calcium from calcium carbonate and calcium citrate is similarly absorbed (around 24%), even if calcium carbonate is less soluble in water. Calcium carbonate is well solubilized in an acidic gastric environment, which can be considered a hydrochloric acid solution. In the presence of sufficient hydrochloric acid, the following reaction will occur:

CaCO_3_ + HCl → CaCl_2_ + H_2_O + CO_2_ (1)

thus, essentially converting calcium carbonate to calcium chloride, with all calcium in ionized form. Part of this calcium is absorbed in the small intestine, but when pH increases again in the intestine, and CO_2_ converts accordingly to HCO_3_^−^ or CO_3_^2−^, this leads to (re)formation of sparingly-soluble calcium carbonate [74]. The importance of acidic solubilization of calcium carbonate is clear from the fact that absorption of calcium carbonate is limited in people with achlorhydria, i.e., those that do not secrete hydrochloric acid; Recker [75] reported that calcium absorption from calcium carbonate was lower in achlorhydric subjects (4.7%) compared to in non-achlorhydric people (22.5%). Interestingly, this situation changed and the calcium absorption rate increased to ~21% when calcium carbonate was consumed by achlorhydric subjects in combination with a standard breakfast, thereby achieving similar calcium absorption levels to people without achlorhydria [75]. This is probably due to the fact that the presence of food delays stomach emptying, thus allowing for better solubilization and dispersion of poorly soluble compounds [76]. Wen and Park [58] reported that gastric emptying time increases from 15 min in a fasting condition to 60 min in fed conditions. On the other hand, calcium citrate is well absorbed both when taken with or without food [76]. Calcium absorption from calcium carbonate (22.5%) and calcium citrate (24.3%) was not significantly different compared with that from skim milk (26%) in non-achlorhydric subjects [75].

Calcium absorption from calcium salts is also affected by particle size. In mice, calcium absorption from calcium carbonate and calcium citrate preparations with smaller particle sizes (average particle sizes 151 ± 19 nm and 398 ± 4 nm, respectively) was higher than from those with larger particle sizes (3773 ± 759 nm and 1793 ± 382 nm, respectively) [77]. In contrast, Elble et al. [78] found in adolescent girls (aged 11 to 14 years) that there was no difference in calcium absorption from a calcium carbonate uptake supplement with small or large particles (13.56 vs. 18 µm). This may be due to the fact that the size difference between the large and small particles in this study was only ~30% [78].

Overall, it is clear that calcium absorption from calcium salts has number of key identifiable factors: i.e., the gastric solubility, particle size but also the presence of other components. Such findings can be considered as the basis for understanding calcium absorption from foods. In addition, methodological aspects should also be considered when studying calcium absorption of food products. This is covered in Section 5.

## 5. Calcium Absorption from Food Products: Methodological Aspects

In subsequent Section 6 and Section 7 of this paper, fractional calcium absorption from different food products is discussed. For this, we considered only data from in vivo studies on humans using an isotopic method. The use of isotopes has greatly improved the accuracy and precision of in vivo nutrient absorption studies [79]. Isotopic methods for the determination of fractional calcium absorption can involve the use of either stable isotopes or radioisotopes, can use either single isotopes or double isotopes and can use intrinsic or extrinsic labeling. Some background of these methods is provided in this section, with the primary aim of facilitating the understanding of fractional absorption data presented in subsequent sections.

As an example of a single stable isotope method, [80] used ^44^Ca to evaluate the absorption of calcium from milk and tofu by fecal recovery. Stool ^44^Ca concentration was measured by mass spectrometry and the percentage of calcium absorption from milk and tofu was from the ^44^Ca excreted in feces, corrected for the baseline, proportional to the ^44^Ca test dose. Another example of a single isotope technique is the evaluation of the blood concentration of the tracer after the administration of the labeled food. For example, Charoenkiatkul et al. [81] used the stable isotope ^44^Ca to label milk, ivy gourd and winged bean. The concentration of ^44^Ca in the blood samples, taken 5 h after the consumption of the labeled test meals, was measured by mass spectrometry and the percentage of calcium absorbed from test meals was calculated as the percentage of the ^44^Ca found in the blood after 5 h, with concentrations of determined ^44^Ca in the blood corrected for height and weight of the subjects to estimate total blood volume. As an alternative to the stable isotope ^44^Ca, it is possible to use a radioisotope, such as ^45^Ca [80]. The administration of a food labeled with a radioisotope allows very precise determination of calcium absorption; however, for e.g., infants, children and pregnant women, the use of radioisotopes is not recommended.

A double isotope method uses two different calcium isotopes. The two tracers can be both stable isotopes, both radioisotopes or one stable isotope and one radioisotope. Usually, one of the two tracers is used to label the test food, while the second is injected intravenously. The calculation of fractional calcium absorption is performed taking into account the dose of isotope administered orally, the dose administered intravenously and the concentration of the two isotopes in the urine completely collected over 24 h after ingestion of the test food [82].

When conducting a calcium absorption study using an isotope technique, the test food can be labeled intrinsically or extrinsically. Intrinsic labeling of a food involves the biological incorporation of the isotope, while extrinsic labeling is obtained by mixing the isotope with food [83]. Various vegetables can be intrinsically labeled by growing the plants hydroponically and adding the isotope to the nutrient solution. Alternatively, it is possible to label the plants during their growth by direct injection or by foliar application [84]. Foods of animal origin can also be intrinsically labeled by adding the isotope to the diet of the animal or by administering it intravenously, intraperitoneally or intramuscularly. The intrinsic labeling of foods allows the fractional absorption of calcium to be determined more precisely, as well as to identify the absorption of calcium from a single food source as part of a meal. However, intrinsic labeling techniques are much more expensive and difficult to implement and cannot be applied to every food material.

For some foods, including milk, the absorption of calcium has also been determined by extrinsic labeling. Extrinsic labeling techniques have been shown to work quite well for liquid foods [85]. Milk, for instance, was mixed with the tracer and then left to equilibrate for 2–16 h before serving [85,86,87,88]. Nickel et al. [85] compared the calcium fractional absorption from bovine milk, labeled both intrinsically and extrinsically. For the intrinsic labeling, a stable calcium isotope was intravenously injected to the cow and the milk was collected over 3 days. Fractional absorption from the intrinsically and extrinsically labeled milks was 0.31 ± 0.09 and 0.32 ± 0.13 (mean ± SEM) respectively, indicating that there was no significant difference (*p* = 0.61). For other foods, such as spinach, it has been shown that extrinsic labeling by blending and mixing with an isotopic tracer is not as valid, as it can lead to overestimating calcium absorption due to an incomplete exchange between the tracer and the endogenous calcium [89].

## 6. Calcium Absorption from Foods

### 6.1. Milk and Dairy Products

Given the importance of milk and dairy products as the primary source of calcium in the human diet (Table 1), it is not surprising that this product category is also the most studied in relation to calcium absorption. Because milk is a product intended naturally for consumption by the mammalian neonate as the sole source of nutrition in the early stages of life, it must be able to deliver sufficient calcium in a bioavailable form from the mother to the neonate, without pathological calcification of the mammary gland [90]. Considering bovine milk as an example, as the most consumed milk, the requirements of calcium and phosphate to sustain healthy skeletal development of the calf far exceed those that can be supplied in soluble form. This is because calcium phosphates are only sparingly soluble salts whose solubility is limited to several millimoles per liter at neutral pH [91]. Bovine milk, however, contains approximately 30 millimoles of calcium and 20 millimoles or inorganic phosphate per liter [92], which is more than an order of magnitude above the solubility of calcium phosphate salts. To enable adequate transport of sufficient amounts of calcium phosphate from the mother to the neonate, some of the calcium is complexed with citrate, which is present in milk at levels up to 10 mM [93,94]. Most importantly, however, is the presence of so-called casein micelles which are protein-based colloids which in bovine milk contain ~70% of total calcium and ~50% of total inorganic phosphate [95]. The calcium phosphate is encapsulated in the casein micelles in the form of small nanoclusters, with a typical diameter of 4–5 nm [96,97]. A casein micelle, which contains several hundred calcium phosphate nanoclusters in addition to tens of thousands of casein molecules, can thus be considered a protein-based carrier for calcium phosphate [96,98]. The calcium phosphate in the casein micelles is in an amorphous form and solubilizes when the pH is reduced; at pH < 5, all calcium phosphate in milk is solubilized [99,100]. In addition to acting as a delivery vehicle for calcium and phosphate for neonates, the casein micelles also play further important roles. Under gastric conditions for humans, they are susceptible to enzymatic coagulation leading to gastric curds and the subsequent phased transit of the casein fraction through the stomach [101]. In addition, the controlled coagulation of casein micelles also forms the basis of the conversion of milk into cheese and yoghurt [102].

**Figure 1:**
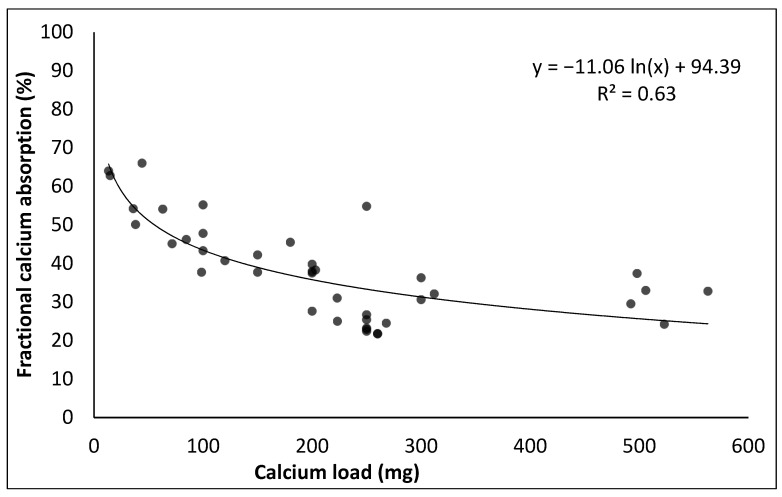
Influence of calcium load on fractional absorption of calcium from dairy products. Data from Table 4.

Factional absorption of calcium from milk as a function of calcium dose, via increasing volume of milk consumed, was studied by Heaney et al. [103], who found a decrease in fractional absorption with increasing calcium dosage. Plotting the natural logarithm of calcium dosage vs. fractional absorption yielded a linear trend [103]. This trend has formed an important basis in comparing different food products, and is widely used. Fractional absorption of calcium from milk, but also various other dairy products, has been the subject of various other studies, as summarized in Table 4. In Figure 1, we show that this relation does not only hold within a single study on a single product, but also across studies and across different dairy products, in addition to milk also including yoghurt and various cheeses. Some deviations can be observed, which may be related to the manner in which the product was ingested (as a single food or as part of a meal) and differences in experimental setup (Table 4).

This trend of exponential decrease in fractional absorption of calcium with increasing intake (Figure 1) yields a number of aspects worth further consideration, i.e., (1) the fact that even at very low intake levels, a fractional absorption of 100% does not appear to be achieved and (2) that the decay in fractional absorption with increasing calcium intake is not linear but log-linear. The first point, i.e., that 100% fractional absorption is not achieved even at very low calcium intake may be related to the fact that in the intestinal conditions in which calcium absorption occurs, not all calcium may be in ionic form. Furthermore, there may be diffusion limitations to the process: i.e., not all calcium may reach the brush border for uptake.

The fact that fractional absorption does not decrease in a linear fashion with calcium intake but in logarithmic fashion can be interpreted in several ways. First and foremost, it should be realized that despite lower fractional absorption, the amount of calcium absorbed with increasing intake increases. In the calcium intake range 10–200 mg (i.e., up to approximately one glass of milk), a good linear relation (R^2^ > 0.99) is found between intake and absorbed amount, but at higher levels of calcium intake, ‘diminishing returns’ are observed.

When considering the absorption of calcium from milk, a number of processes should be considered. In the oral phase of the digestion process, the milk and its salt balance remain unchanged. However, when milk enters a fasting stomach, it comes into contact with a small amount (e.g., ~50 mL) of gastric juice, which will have a pH between 1 and 2 for an adult [101]. However, given the large volume of milk compared to gastric juice (assuming consumption of a glass of milk on a fasting stomach) and the buffering capacity of the milk, gastric pH will rise rapidly, to values > 6 [121]. At this point, the milk in the stomach is somewhat diluted and slightly acidified, as a result of which some of the calcium phosphate that was present in the casein micelles will solubilize [99,100]. Following this, gastric juice will be secreted into the stomach, which will reduce pH gradually [121]. In addition, the pepsin in the gastric juice will hydrolyze κ-casein, resulting in aggregation of casein micelles to form gastric curd [101]. This process is observed to occur already at pH values > 6 [101] and can lead to curd particles with sizes exceeding several millimeters, i.e., at least 4 orders of magnitude larger than the original casein micelles [122,123]. This thereby notably increases diffusion distances for acid into the particles and solubilized calcium and phosphate from the particles to the surrounding serum, and thereby slows down the rate of solubilization of calcium phosphate. The fact that enzymatic coagula of milk are known to synerese [124,125] further hampers the influx of acid, thereby hindering acidification and calcium solubilization from the coagula. The contraction of the curd matrix as a result of syneresis will also reduce the speed of dissociation [126]. For cheese, which is also a concentrated para-casein matrix, it has been shown that that the diffusion coefficient of salt is 4–5 orders of magnitude smaller than in water [127], indicating that further impairment of diffusion of salts and acid into and from the curd particles. Hence, while gastric pH values < 5 should theoretically be sufficient to solubilize all calcium and phosphate from the casein micelles [99,100], the enzymatic coagulation of the micelles will hinder this process and will delay the release calcium and phosphate from the gastric curd particles. This delayed release can ultimately have benefits for calcium absorption due to reduced concentrations of calcium in the gastric phase that transits to the stomach. Considering an initial volume of 50 mL, gastric juice and consumption of 250 mL milk with a calcium content of 30 mmol/L and an inorganic phosphate content of 20 mmol/L, complete solubilization of all calcium and phosphate will lead to concentrations of ~25 and 17 mmol/L for calcium and phosphate, respectively. However, as outlined above, dilution with gastric fluid and slow release from gastric coagula will notably reduce calcium phosphate concentrations in the digesta leaving the stomach. From buffering curves of milk [128], it can be estimated that the amount of gastric fluid required to acidify 250 mL of milk to pH 2 is approximately equal to the milk volume, thus notably reducing concentrations of soluble calcium and phosphate in the gastric phase to ~15 and 10 mmol/L; as gastric emptying time for a glass of milk is several hours [101], the calcium load leaving the stomach would be in the range of several millimoles per hour, which may be considered a controlled release, thus ensuring adequate uptake of calcium. The rate of gastric emptying is determined by a number of factors, including restrictions on volume flow, caloric density, pH and rheological properties [129].

An interesting perspective, in this respect, is gained from the data by Fairweather-Tait et al. [115] who compared fractional absorption of calcium from skimmed milk and Ca-enriched skimmed milk and noted that the former had a notably higher (45.5%) fractional absorption than the latter (35.7%). This may be explained based on the fact that subjects ingested a smaller volume of the Ca-enriched product (83 mL) compared to the milk (136 mL) for the same Ca intake and thus reduced gastric volume [115]. Furthermore, the use of Ca-gluconate in the preparation of the Ca-enriched milk would have a notable effect on buffering. As a result of the reduced intake of milk for the Ca-enriched product, the amount of protein, phosphate and citrate, the main buffering compounds in milk [128], is also reduced by ~40%. This reduction in buffering is only compensated to a small extent by the addition of Ca-gluconate as a calcium source. Gluconate is only a weak buffer with a pKa < 4. Hence, much less buffering would be observed when this product enters the stomach than for milk, leading to a lower pH upon mixing with the gastric fluid present in the fasting stomach. This would lead to a lower pH and consequently more solubilization of micellar calcium phosphate before pepsin-induced curd formation would commence, in addition to the already higher levels of soluble Ca in the fortified milk than control milk. As a result, fractional absorption of calcium from the aforementioned fortified milk was lower than for the control milk [115], presumably due to more rapid gastric release of calcium into the intestine, thereby reducing overall absorption. For milk fortified with an insoluble calcium source, i.e., tricalcium phosphate, Lopez-Huertas et al. [120] actually observed significantly higher fractionation absorption than from control milk (27.5 vs. 24.5%). This can be related to the fact that tricalcium phosphate is only sparingly soluble and more slowly solubilized than calcium phosphate inside the casein micelles (Huppertz, unpublished data), as a result of which the controlled release of calcium into the intestine is achieved. These studies highlight the importance of careful consideration of calcium fortification of milk, as well as other dairy products, to ensure maximal fractional absorption.

For other dairy product types, e.g., cheese and yoghurt, similar matrix effects may be considered. Particularly interesting, in this respect, is the study of Nickel et al. [85] comparing cheddar cheese and yoghurt and showing a notably higher fractional absorption of calcium from the former (37.4 vs. 24.2%). This difference can probably also be attributed to physicochemical properties and equilibria during the gastric phase. Both cheese and yoghurt have a notably lower pH and higher acidity than milk, but differ strongly in structure. Cheese may be considered a highly concentrated gelled casein matrix whereas yoghurt is also gelled (if set yoghurt is used) or a suspension of microgel particles (if stirred yoghurt is used) but the gel is much less concentrated [130]. Neither yoghurt or cheese are prone to coagulation under gastric conditions [131,132,133], unlike milk, and release of calcium would thus depend on (1) release of calcium from the gelled structure and (2) breakdown and gastric emptying of the structure. Interesting similarities may be drawn to postprandial aminoacidemia for these products. In a recent study, Horstman et al. [133] showed that postprandial amino acid levels in blood increased more quickly and to higher levels, but also decreased much more rapidly on consumption of (stirred) yoghurt compared to cheese, with the latter showing a much more gradual release. As outlined above for milk, this more gradual and sustained release into the intestine is assumed to be beneficial for increasing fraction absorption of calcium from cheese. Hence, although cheese does not coagulate further in the stomach, the fact that it is a highly concentrated product matrix with notable buffering capacity in which mobility is low benefits gradual release of calcium to the intestine, where the matrix can be further broken down by proteases, and the calcium and other components from the matrix are released. In contrast, the much weaker gels and small gel particles in yoghurt, combined with the fact that most of the calcium is already in soluble form at the pH of yoghurt (pH 4.0–4.5) [134] and pH will decrease further in the gastric phase of digestion, which can lead to much more rapid release of calcium into the intestine and therewith lead to reduced fractional absorption. It is, in this perspective, it is thus very important to keep in mind that high levels of soluble calcium in the product are not by definition beneficial for fractional absorption. Static in vitro methods focusing solely on this topic should thus be interpreted with care.

### 6.2. Non-Dairy Products

As outlined above, most of the dietary calcium in many countries comes from dairy products, but some non-dairy products can also contribute notably, e.g., vegetables, starchy foods, dried fruits and water. However, to be considered a suitable source of calcium, it is important that foods not only contain it in adequate quantities, but also that the calcium is also sufficiently bioavailable. Some non-dairy foods are characterized by a lower fractional absorption of calcium, despite containing it in high quantities. In several foods of plant origin, for instance, the absorption of calcium and other minerals is adversely affected by the presence of anti-nutritional factors, such as oxalates and phytates [135].

Table 5 shows the data from different studies on the absorption of calcium in humans from different non-dairy foods. Particularly spinach (American and Chinese varieties) and rhubarb, rich in calcium, are characterized by a very low fractional absorption (5.1%, 9.3% and 9.2% respectively; Table 5).

This may probably be due to the high content of oxalic acid in these products because calcium oxalate is a very insoluble salt [108,139]. Calcium oxalate is insoluble at a neutral or alkaline pH, and even at pH 2 has only very low solubility (Table 6).

Insoluble calcium oxalates, precipitates and is eliminated with the feces but according to Hanes et al. [141], a minimal part of calcium oxalate could be absorbed intact. Instead, the part of the soluble oxalate not bound with calcium is absorbed into the large intestine (colon) through a passive pathway [142]. Although some studies reported that the main site where the absorption of free oxalate is the small intestine [143]. Absorbed soluble oxalate may still bind calcium present in the blood vessels, and precipitate in the kidney or urinary tract leading to the formation of kidney or urinary tract stones [144]. The negative effect of oxalates on calcium absorption increases when the molar ratio of total oxalate: calcium is major than 9:4 and this occurs in spinach and rhubarb [145]. Chai and Liebman [146] suggested that the boiling method is effective in reducing total oxalate (soluble and insoluble) content in spinach and rhubarb stalks compared to steaming (Table 7).

In contrast, fractional calcium absorption is much higher in vegetables which are low in oxalate, e.g., kale, broccoli, bok choy, kai choy and choy sum (Table 5; [105,136]). In addition to oxalates, calcium absorption can also be inhibited by phytates, but to a lesser extent [147]. The inhibitory effect of phytate on calcium absorption was confirmed also by Heaney et al. [106], who found that calcium absorption from low-phytate soybeans was significantly higher than that from high-phytate soybeans (41.4 vs. 31.0%). Furthermore, calcium absorption was higher from tortillas with low phytate content than from their counterparts with regular phytate content (50 vs. 35%; [138]). In rats, phytate content did not affect calcium absorption from intrinsically labeled soybeans [148], but this discrepancy is likely related to the fact that rats have an intestinal phytase activity that is around 30 times higher than that of humans [149].

Phytic acid is a phosphorus-based substance, which is found in cereals, legumes, nuts and seeds, but the highest phytate content was found in soybean (~2 g/100 g) [88,144]. Phytic acid can bind calcium in the stomach (below pH 4), forming a soluble calcium-phytate complex [150]. During the passage through the stomach and small intestine, calcium phytate showed a rapid decrease in solubility due to the increase in pH, and precipitates reducing its absorption [151]. When phytate reaches the colon, it is degraded by phytase enzyme produced by the gastrointestinal microflora, this process depends on calcium level in the diet [152]. Grynspan and Cheryan [150] reported that maximum calcium precipitation (~100%) occurs only when the molar ratio of phytate:calcium is 4:1 and pH ˃ 8. Boiling and cooking processes have only slightly effects in reducing phytate, as it is thermally stable below 150 °C [153].

The influence of phytate was also clear for wheat flour products, which showed higher calcium absorption than wheat bran products, which are rich in phytate [86]. Data reported in Table 5 show also that foods with low amounts of both phytate and oxalate, such as cassia leaves (42.6%), winged beans (39.1%) and the ivy gourd (47.6%) have a high calcium absorption [81,114]. Mineral water has been shown to be a source of absorbable calcium [110,118] but concentrations are relatively low, so this would need considerable amounts of consumption to make meaningful contributions to overall calcium intake.

### 6.3. Calcium-Fortified Foods

Calcium-fortified foods are used all over the world and can help people to fill calcium gaps in their daily diets. Table 8 contains a list of studies on calcium absorption in which various calcium fortified foods were compared with bovine milk that was not fortified. A wide variety of calcium compounds, such as calcium citrate, calcium malate, calcium carbonate, tricalcium phosphate, calcium gluconate, calcium chloride and calcium sulfate, were used as food additives. It can be observed that calcium absorption from soy drink fortified with tricalcium phosphate was lower than that of bovine milk [87]. However, when the soy drink was fortified with calcium carbonate, the absorption of calcium was similar to that of bovine milk (21.1 vs. 21.7%) and both were significantly higher (*p* < 0.05) than that of the soy drink fortified with tricalcium phosphate (18.1%), with the same calcium load [88].

Weaver et al. [80] studied the absorption of calcium from tofu fortified with calcium chloride and calcium sulfate in Caucasian and Asian populations. The results show that, in both studies, the calcium absorption from fortified tofu was similar to that of milk. In a study carried out by Martin et al. [112], bread fortified with calcium sulfate was tested for calcium absorption in comparison with bovine milk. Calcium absorption was determined in 18 healthy women who ingested a serving of bread containing 300 mg of calcium in a randomized crossover design using a cup of milk as control. It was found that calcium sulfate fortified bread was slightly better absorbed than milk (43.02 vs. 36.27%). Pak et al. [154] studied calcium absorption in orange juice fortified with either calcium citrate or calcium malate in 16 healthy individuals and found that calcium absorption was similar in both orange juices (40.1 vs. 40.6%). Furthermore, the absorption of calcium from calcium sulphate-fortified mineral water was investigated and found to be similar to that from bovine milk [111]. On the other hand, Hawthorne et al. [109] used an innovative strategy to increase calcium absorption in carrots, given their low calcium content. Biotechnology-modified carrots containing high levels of calcium have been developed. The results showed that the absorption of calcium from biotechnologically modified carrots was slightly lower than in milk, even though the portions of modified carrots were too large to be considered as a substitute source for milk.

## 7. Selection of Calcium Sources for Health and Sustainable Diets

As outlined previously, various considerations can form the basis for inclusion of food products as dietary sources of calcium. Calcium content and calcium bioavailability are important aspects to consider from a nutritional aspect. However, consideration of the environmental impact of the production of the food items is also important to ensure that products fit in sustainable diets and do not cause an environmental impact that is disproportional to the nutritional contribution of the product [155,156]. To explore this aspect, a database of 218 Dutch food items was used for which LCA data were released by National Institute for Public Health and the Environment (RIVM) in the Netherlands [157]. Food composition data for these food items were attained from the Dutch food composition database also released by RIVM [158]. For each food item, the amount of product that needed to be consumed to meet 20% of the RDI for calcium in the Netherlands of 1000 mg was calculated, as well as the greenhouse gas (GHG) emissions, expressed in CO_2_-eq, with this amount of product.

Results are shown in Table 9, with only food items shown that could make at least a proportional contribution to the diet on an energy basis (i.e., Ca/energy > 1000 mg/2250 kcal). This yielded a selection of 60 food items (i.e., just over 25% of all products), of which 21 fell in the category of dairy products (including cheese) and 18 in the category of vegetables, together spanning almost two thirds of the group. Next to this, the selection included some fruits (*n* = 7) and fish (*n* = 6) and various other products. For all fruit products, however, amounts required to achieve 20% of RDI were unrealistically high, with the lowest amount being 111 g of dried figs per day, even increasing to 1200 g of strawberries per day. Likewise, for all fish products, intake of >120 g per day was required for 20% of RDI for Ca, is unrealistically high on a daily basis. While these products will of course contribute to overall calcium intake, they are unlikely to be the cornerstones of calcium intake in the diet.

For the vegetables, required intake for kale (78 g) and spinach (111 g) appear more reasonable, but for the latter, it should be remembered that bioavailability of Ca is very low (Table 5, [108]. For milk and dairy products, serving size for almost all products (15 out of 21) to achieve 20% of RDI for Ca could be considered to be single portions, e.g., up to 200 g of milk or yoghurt and up to <25 g of cheese. The only exceptions in this respect were custard-type products, ice cream, cheese spread, mozzarella cheese and fresh goat’s cheese. Another good source of Ca was a vegetarian schnitzel (Table 9), which is actually a dairy-based product.

When considering greenhouse gas (GHG) emissions, expressed as CO_2_-eq for the portion of each food item required to provide 20% of RDI for Ca, values ranged from 0.09 kg CO_2_-eq for seaweed kelp to 7.69 kg CO_2_-eq for strawberries (Table 9). In general, it is clear that mainly calcium-rich foods have lower CO_2_-eq per 20% RDI for Ca. Again here, dairy products, kale, lettuce and the (dairy-based) vegetarian schnitzel appeared to be the most apparent choices for making a notable impact on calcium intake without excessive impact on GHG emissions. While seaweed kelp and linseed are also apparent sources of Ca with comparatively low CO_2_-eq, the required levels of consumption for a 20% contribution to RDI for Ca appear unrealistically high, and even exceeded boundaries for safe consumption of products for linseed because iodine intake calculated from this portion would notably exceed the upper intake level recommended by EFSA (i.e., 600 μg per day for adults).

As such, the fact that at present almost 60% of all dietary calcium in a Dutch diet comes dairy products [159] appears logical. This class of products makes large contributions to the RDI of calcium even with single portions (i.e., a glass of milk, a bowl of yoghurt or a slice of cheese). Furthermore, from a perspective of environmental impact, however, dairy appears to be the logical choice, with GHG emissions per unit of Ca for dairy products lower than for most other food items that could be considered credible calcium sources based on the criterium of supplying at least 1000 mg of Ca per 2250 kcal.

Of course, food items should not be judged on single nutrients (i.e., in this case Ca), as virtually all food items are sources of multiple nutrients. To illustrate this, the food items included in Table 9 were also considered in terms of nutrient richness. For this purpose, we explored an additional 21 nutrients and if the portion size identified in Table 9 as required for providing 20% of RDI of Ca also made substantial contributions to RDI of other nutrients. Results are shown in Supplementary Table S1 and highlight some interesting aspects.

For most dairy products, portions required to provide 20% of RDI for Ca provided at least 20% of RDI for one other nutrient, i.e., phosphorus. In fact, for virtually all food items, phosphorus was provided in at least similar proportion of RDI as Ca. For other nutrients, such abundance among food products could not be seen, although dairy products were also shown to be rich sources of vitamin B2 and vitamin B12. For iron, not a single product listed in Supplementary Table S1 could provide 20% of RDI in the portions outlined in Table 9 and for vitamin D, only the fish products were shown as main contributors (Supplementary Table S1).

## 8. Conclusions

Calcium is an essential nutrient in the human diet, and dietary intake of calcium does not meet recommendations in many parts of the world. In areas of the world where dietary calcium intake does meet recommendations, dairy consumption makes the largest contribution to calcium intake. This important contribution can be made due to the fact that in a single serving, dairy products provide high levels of calcium which become bioavailable under the conditions in the gastrointestinal tract. For other food groups, the presence of phytate and oxalate can strongly limit calcium absorption because the complexes are not solubilized and ionized even at the low pH encountered during gastric transit. In addition, controlled gastric release can also enhance calcium absorption. Hence, calcium fortification strategies which do not consider this aspect run the risk of increasing dietary calcium but not absorbable calcium. Overall, consideration of calcium in food matrices and changes therein during digestion is crucial for enabling optimal utilization of dietary calcium.

## Figures and Tables

**Table 1:** Average daily calcium intake for adults and the main dietary sources of calcium in different countries.

Country	Calcium Intake (mg/day)	Ages (Years)	Main Food Sources of Calcium	References
Greece	1039	35–74	Dairy products, cereals and products, vegetables	[11]
The Netherlands	1033	35–74	Dairy products, cereals and products, beverages (non-alcoholic)	[11]
Denmark	1011	35–74	Dairy products, cereals and products, beverages (non-alcoholic)	[11]
Canada	973	≥25	Milk and dairy products	[12]
Spain	972	35–74	Dairy products, cereals and products, beverages (non-alcoholic)	[11]
United Kingdom	969	35–74	Dairy products, cereals and products, cakes	[11]
Sweden	955	35–74	Dairy products, cereals and products, beverages (non-alcoholic)	[11]
Germany	942	35–74	Dairy products, cereals and products, beverages (non-alcoholic)	[11]
USA	934	≥19	Dairy products, vegetables, fruits	[13,14]
France	918	35–74	Dairy products, beverages (non-alcoholic), vegetables	[11]
Italy	808	35–74	Dairy products, cereals and products, vegetables	[11]
Australia	672	14–25	Regular milk, cheese, bread, low-fat milk	[15]
India (metropolitan)	526	43	Cereals and products, dairy products, vegetables	[16]
China	369	18–64	Vegetables, legumes, cereals and products	[10]
India (urban)	308	47	Cereals and products, dairy products, vegetables	[16]
India (rural)	269	40	Cereals and products, vegetables and dairy products	[16]

**Table 2:** Transit time in segments of the intestine in humans and rats.

	Humans ^1^	Rats ^2^
	Transit Time (min)	Transit Time (min)
Duodenum	15.6	3
Jejunum	102	43
Ileum	78	141
Total	195.6	187

^1^ Data from [58]. ^2^ Data from [57].

**Table 3:** Calcium content, pH and solubility of various calcium salts.

Calcium Salts	Calcium Content (%)	pH	Solubility (g/L)	References
Calcium lactate	13	not reported (water)	66	[72]
Calcium gluconate	9.0	not reported (water 21 °C)	30	[67]
Calcium carbonate	40.0	4.5	46	[67,68]
		6.0	3.8	[68]
		7.5	0.13	[68]
		8.5 (water)	<0.005 ^a^	[68]
		not reported (water 21 °C)	0.014	[67]
Calcium citrate	21	2.0	17	[68,72]
		4.5	0.33	[68]
		5.6 (water)	0.23	[68]
		6.0	0.20	[68]
		7.5	0.23	[68]
Calcium phosphate	17–36	2.0	21	[68,72]
		4.5	0.63	[68]
		6.0	0.079	[68]
		6.3 (water)	0.037	[68]
		7.5	0.019	[68]
		not reported (water)	slightly soluble	[72]
Calcium lactate citrate	16.2	not reported (water 21 °C)	98	[67]
Calcium lactate malate	18.1	not reported (water 21 °C)	115	[67]

^a^ Below the limit of quantification.

**Table 4:** Fractional absorption of calcium from dairy products.

Food	Serving Size (g or mL)	Calcium Load (mg)	Fractional Calcium Absorption (%) ^a^	Meal Composition	Isotopic Method ^b^	References
Milk	DNS	71.6	45.1 (1.8)	Milk was ingested with one or two slices of low-calcium Italian bread toasted and served with butter, as well as coffee or tea, with artificial sweetener if desired	DNS	[104]
Milk	DNS	200	37.9 (2.6)	Milk was ingested as a part of a neutral breakfast consisting of two pieces of white bread toast with butter or margarine and coffee after an overnight fast	R/E	[105]
Milk		63	54.1 (4.5)			
Milk		120	40.7 (2.1)			
Milk 2% fat	DNS	98.5	37.7 (1.4)	Milk was ingested in the middle of a neutral meal consisting of two pieces of white bread toast with butter or margarine as well as coffee or tea	R/E	[106]
Milk	DNS	312	32.1 (2.7)	Milk was ingested in the middle of a neutral meal consisting of two pieces of white-bread toast with butter or margarine and coffee, tea or diet cola (without sugar)	R/E	[107]
Milk	DNS	200	27.6	Milk was ingested as a part of a standard breakfast consisting of two pieces of toasted white bread with butter or margarine and a cup of coffee or tea (with artificial sweetener)	DR/E	[108]
Milk low fat	30 mL	38	50.1 (3)	Milk was ingested with 170 g calcium fortified orange juice in order to provide approximately 300 mg calcium	SSI/E	[109]
Milk	90.3 g	100	43.3	Water was consumed midway through a light breakfast consisting of low-calcium white bread, toasted, with butter and tea or coffee (with artificial sweetener, if desired)	R/E	[110]
Milk (2.8 g fat/L)	200 mL	223.2	25.0 (2.2)	200 mL of milk was ingested with 300 mL distilled water after an overnight fast	DSI/E	[111]
Milk	A cup of milk	300	36.3 (2.2)	Milk was ingested as a part of a standard test breakfast consisting of two pieces of toasted white bread with butter or margarine and a cup of coffee or tea (with artificial sweetener, if desired). One of the two pieces of bread was calcium fortified	R/E	[112]
Milk 2% fat	240 mL	260	21.7 (0.9)	Milk was ingested with a slice of calcium-free toast	DSI/E	[88]
Milk	6.9 g	13.3	64 (2.9)	Milk was ingested without meal	R/E	[86]
Milk	140.1 g	200	37.5 (1.7)			
Skimmed milk	208 g	260	21.8 (1.4)	208 g of milk was ingested together with 120 g white wheat bread, 27 g unsalted butter and 100 g ultrapure water	R/E	[113]
Milk 2% fat	67.3 g	250	54.8 (4)	Milk was ingested with breakfast (not specified)	SSI/E	[80]
Skim milk	DNS	200	39.8 (3)	Milk was ingested with two slices of low-calcium Italian bread toasted and served with butter as well as coffee or tea (with artificial sweetener, if desired)	R/E	[80]
Milk	92.4 g	100	55.2 (2.7)	Milk was served as part of breakfast after an overnight fast with 100 g cooked rice	SSI/E	[81]
Whole milk	DNS	100	47.8 (2.9)	Milk was ingested with 90 g of cooked white rice	TSI/E	[114]
Skimmed milk	136 mL	180	45.5 (1.9)	Milk was ingested without meal	DSI/E	[115]
Milk 2% fat	DNS	300	30.6 (1.5)	Milk was ingested as part of a breakfast after an overnight fast with three pieces of low-calcium Italian-style white bread toasted with butter a cup of coffee (with artificial sweetener if desired)	R/E	[87]
Milk	20 mL	44	66 (0.1)	Milk was ingested without meal, but the ingestion was followed immediately by 200 mL of distilled water	R/E	[116]
Milk 2% fat	240 mL	14.7	62.8	Milk was taken in the middle of a light breakfast consisting of two pieces of toasted Italian bread with butter, together with coffee (decaffeinated or regular) or tea	DSI/E	[103]
Milk 2% fat	240 mL	35.9	54.2			
Milk 2% fat	240 mL	84.6	46.2			
Milk 2% fat	240 mL	203.1	38.3			
Milk 2% fat	240 mL	492.4	29.5			
Milk	488 g	563	32.8 (4.0)	Test foods were ingested with breakfast (not specified) after an overnight fast	SSI/I	[85]
Cheddar cheese	108 g	498	37.4 (9.2)			
Processed cheese	145 g	506	33.0 (4.3)			
Yoghurt	481 g	523	24.2 (3.4)			
Whole milk	DNS	250	26.7 (2.5)	DNS	DR/E	[117]
Chocolate milk		250	23.2 (1.8)			
Yoghurt		250	25.4 (2.9)			
Imitation milk		250	22.4 (1.8)			
Cheese		250	22.9 (1.7)			
Fresh cheese	208 g	150	42.2 (3.3)	Test meals were ingested without meal	DSI/E	[118]
New fresh cheese	94 g	150	37.7 (2.9)			
Milk 1.7% fat	200 g	223	31 (2.3)	Milk was ingested within five minutes after the standardized breakfast which consisted of two slices of white bread with margarine and strawberry jam and 125 mL mineral water	DSI/E	[119]
Milk 1.9% fat	165–224 mL	268	24.5 (1.9)	Test foods were ingested in the middle of a light breakfast consisting of 2 slices of toasted white bread with butter and jam	DSI/E	[120]

^a^ Values are means, with SEM between brackets. ^b^ E, extrinsically; I, intrinsically; R, radioisotope; DR, double radioisotope; SSI, single stable isotope; DSI, dual stable isotope; TSI, trial stable isotope; DNS, data not supplied.

**Table 5:** Fractional absorption of calcium from non-dairy food products.

Foods	Serving Size (g)	Calcium Load (mg)	Fractional Calcium Absorption (%) ^a^	Meal Composition	Isotopic Method ^b^	References
Broccoli	DNS	82.4	47.8 (3.0)	Cooked and pureed vegetables were fed as a part of breakfast consisting of two slices of low-calcium Italian bread, toasted and served with butter as well as coffee or tea, with artificial sweetener, if desired	R/I	[136]
Bok choy stems		83.0	51.9 (3.6)			
Bok choy leaves		83.0	52.0 (1.9)			
Kale		83.0	52.7 (2.4)			
White beans	DNS	71.6	22.5 (1.5)	Cooked and pureed beans were ingested with one or two slices of low-calcium Italian bread, toasted and served with butter as well as coffee or tea, with artificial sweetener, if desired	R/I	[104]
Red beans		71.6	19.3 (1.3)			
Pinto beans untreated		71.6	23.1 (1.4)			
Pinto beans phytase treated		71.6	31.8 (2.2)			
Kai Choy (Chinese mustard greens)	144	200	39.9 (2.1)	Cooked and pureed vegetables were fed as a part of a neutral breakfast consisting of two pieces of white bread toast with butter or margarine and coffee after an overnight fast	R/I	[105]
Choy Sum (Chinese cabbage flower leaves)	127	200	40.2 (1.7)			
Chinese spinach	119	200	9.3 (0.7)			
Sweet potatoes	115	63	22.8 (2.1)			
Rhubarb	58	120	9.2 (0.8)			
Soybean high phytate Soybean low phytate	88 88	97.8 99.2	31.0 (1.8) 41.4 (1.9)	Cooked soybeans were ingested as breakfast after an overnight fast and accompanied by coffee or tea (with artificial sweetener if desired)	R/I	[106]
Kale	150	288	40.9 (3)	Cooked and pureed kale were ingested in the middle of a neutral meal consisting of two pieces of white-bread toast with butter or margarine and coffee, tea or diet cola (without sugar)	R/I	[107]
Spinach	DNS	200	5.1	Cooked and pureed spinach were administered as a part of a standard breakfast consisting of two pieces of white bread toast with butter or margarine and coffee or tea (with artificial sweetener if desired) after an overnight fast	DR/I	[108]
Wheat bread	78.3	13.3	81.7 (3.7)	Bread was served with butter Wheat bran cereal was served with milk	R/I	[86]
Wheat Bran cereal	44.5	200	22.3 (1.1)			
Wheat bread (crust removed)	61.0	10.4	70.3 (2.8)			
Light cookies	111.0	18.8	64.8 (2.2)			
Dark cookies	101.4	20.4	65.4 (2.7)			
Winged beans	400	110	39.1 (2.9)	The test foods were served as part of breakfast with 100 g cooked rice after an overnight fast	SSI/E	[81]
Ivy gourd	150	114	47.6 (2.5)			
Cassia	142	100	42.6 (2.8)	Cooked cassia was ingested with 90 g of cooked white rice	TSI/E	[114]
Rice based cereal	DNS	481	16.0 (1.2)	Test foods were ingested with together with 250 mL water	R/E	[137]
Whole grain cereal		541	17.0 (1.0)			
Tortillas, maize with typical-phytate content	140	140	35 (3.1)	Tortilla meals were ingested without meal	DSI/E	[138]
Tortillas, maize with low-phytate content	140	140	50 (1.3)			
Sangemini	294.4	100	47.5	Water was consumed midway through a light breakfast consisting of low-calcium white bread, toasted, with butter, and tea or coffee (with artificial sweetener, if desired)	R/E	[110]
Ferrarele	340	150	37.0 (2.8)	Water was ingested without meal	DSI/E	[118]
Freeze-dried small Bengali fish	6.28	280	23.8 (1.3)	6.28 g of fish was ingested together with 120 g white wheat bread, 27 g unsalted butter and 308 g ultrapure water	R/E	[113]

^a^ Values are means, with SEM between brackets. ^b^ E, extrinsically-labeled; I, intrinsically-labeled; R, radioisotope; DR, double radioisotope; SSI, single stable isotope; DSI, dual stable isotope; TSI, trial stable isotope; DNS, data not supplied.

**Table 6:** Solubility of calcium oxalate at different pH values.

pH	Calcium Oxalate Solubility (g/L)	References
2.0	0.011	[68]
4.5	<0.005 ^a^	[68]
6.0	<0.005 ^a^	[68]
7.5	<0.005 ^a^	[68]
8.5 (water)	<0.005 ^a^	[68]
8.5 (water)	0.0061	[140]

^a^ Below the limit of quantification.

**Table 7:** Influence of steaming and boiling on the total oxalate (soluble and insoluble) content in spinach and rhubarb.

	Total Oxalate (mg/100 g of Wet Weight) ^a^			
	Raw	Steamed	Boiled	Cooking Time (min)
Rhubarb stalks	532 ± 8	505 ± 2	309 ± 7	15
Spinach	1145 ± 33	797 ± 12	460 ± 9	12

^a^ Values are expressed as mean ± SD; data from [146].

**Table 8:** Fractional absorption of calcium from calcium-fortified food products.

Foods	Serving Size (g or mL)	Calcium Load (mg)	Fractional Calcium Absorption (%) ^a^	Meal Composition	Isotopic Method ^b^	References
Orange juice with calcium citrate	180 mL	300	40.1 (2.1)	Orange juices were ingested without meal	R/E	[154]
Orange juice with calcium malate	180 mL	300	40.6 (2.2)			
Milk 2% fat	240 mL	260	21.7 (0.9)	Foods were ingested with a slice of calcium-free toast	DSI/E	[88]
Soy drink with calcium carbonate	240 mL	260	21.1 (1.3)			
Soy drink with tricalcium phosphate	240 mL	260	18.1 (0.9)			
Milk	A cup of milk	300	36.27 (2.2)	Foods were ingested as a part of a standard breakfast consisting of two pieces of toasted white bread with butter or margarine and a cup of coffee or tea (with artificial sweetener, if desired). One of the two pieces of bread was calcium fortified	R/E	[112]
Bread with calcium sulphate	16.8 g	300	43.02 (2.5)			
Medium fat milk (2.8 g fat/L)	200 mL	223.2	25.0 (2.2)	Milk was ingested with 300 mL distilled water after an overnight fast	DSI/E	[111]
Mineral water with calcium sulfate	500 mL	224	23.8 (1.6)	Water was ingested without meal		
Milk 2% fat	67.3 g	250	54.8 (4)	Test foods were ingested with breakfast (not specified)	SSI/E	[80]
Tofu with calcium chloride	DNS	250	49.3 (5.6)		SSI/E	
Skim milk	DNS	200	39.8 (3)	Test meals were ingested at breakfast consisting in two slices of low-calcium Italian bread, toasted and served with butter as well as coffee or tea (with artificial sweetener, if desired)	R/E	[80]
Tofu with calcium sulfate	DNS	200	39.0 (3.5)		R/E	
Milk 2% fat	DNS	300	30.6 (1.5)	Foods were ingested as part of a breakfast after an overnight fast with three pieces of low-calcium Italian-style white bread toasted with butter a cup of coffee (with artificial sweetener if desired)	R/E	[87]
Soy drink with tricalcium phosphate 2% fat	DNS	300	35.8 (1.67)		R/E	
Milk low fat	30 mL	38	50.1 (3)	Milk and carrots were ingested with 170 g calcium fortified orange juice in order to provide approximately 300 mg calcium	SSI/E	[109]
Ca-enriched carrots	65 g	40	42.6 (2.8)		SSI/I	
Milk 1.9% fat	165–224 mL	268	24.5 (1.9)	Test foods were ingested in the middle of a light breakfast consisting of 2 slices of toasted white bread with butter and jam	DSI/E	[120]
Milk with tricalcium phosphate	165–224 mL	268	27.5 (2.0)			
Skimmed milk	136 mL	180	45.5 (1.9)	Ingested without meal	DSI/E	[115]
Skimmed milk with calcium gluconate	83 mL	184	35.7 (4.7)			
Milk 1.7% fat	200 g	223	31 (2.3)	Test foods were ingested within five minutes after the standardized breakfast which consisted of two slices of white bread with margarine and strawberry jam and 125 mL of mineral water	DSI/E	[119]
Ice cream 3% butterfat	60 g	227	26 (2.0)			
Ice cream 9% coconut oil	60 g	224	28 (1.3)			
Milk	20 mL	44	66 (0.1)	Test foods were ingested without meal, but the ingestion was followed	R/E	[116]
Soy drink calcium-fortified	20 mL	44	65 (0.1)	immediately by 200 mL of distilled water		

^a^ Values are means, with SEM between brackets. ^b^ E, extrinsically-labeled; I, intrinsically-labeled; R, radioisotope; DR, double radioisotope; SSI, single stable isotope; DSI, dual stable isotope; DNS, data not supplied.

**Table 9:** Calcium content (in mg/100 g product and mg/2250 kcal), greenhouse gas emissions (GHG; in kg CO_2_ equivalents per 100 g product and per 200 mg calcium) and product quantity required to obtain 200 mg calcium for selected Dutch food items. Only food items containing at least 1000 mg calcium per 2250 kcal are included.

Food Item	Food Category	Ca (mg/100 g) ^a^	GHG (kg CO_2_-eq/100 g) ^b^	Ca (mg/2250 kcal) ^a^	Product (g/200 mg Ca) ^c^	kg CO_2_-eq per 200 mg Ca ^c^
Seaweed kelp, raw	Miscellaneous	168	0.09	8043	119.0	0.10
Flax seed	Nuts and seeds	255	0.17	1203	78.4	0.13
Kale	Vegetable	231	0.16	15750	86.6	0.14
Vegetarian schnitzel	Meat/dairy substitutes	800	0.59	9574	25.0	0.15
Cheese, 20+	Dairy	1059	1.04	9686	18.9	0.20
Cheese Edam, 40+	Dairy	896	1.10	6222	22.3	0.25
Lettuce, medium	Vegetable	53	0.07	7950	377.4	0.26
Herring in tomato sauce	Fish	150	0.20	2021	133.3	0.26
Spinach, frozen	Vegetable	162	0.22	15188	123.5	0.28
Milk, butter	Dairy	109	0.15	8175	183.5	0.28
Lettuce, crop	Vegetable	49	0.07	8481	408.2	0.28
Yogurt, low-fat	Dairy	152	0.22	9243	131.6	0.29
Root	Vegetable	27	0.04	1841	740.7	0.30
Onion	Vegetable	29	0.04	1764	689.7	0.30
Milk, skim	Dairy	126	0.20	8100	158.7	0.31
Cheese Gouda,48+	Dairy	816	1.31	4976	24.5	0.32
Yogurt, whole	Dairy	143	0.24	5547	139.9	0.33
Milk, semi-skimmed	Dairy	123	0.20	6150	162.6	0.33
Yogurt, semi-skimmed	Dairy	139	0.23	6255	143.9	0.33
Yogurt, low-fat with fruits	Dairy	129	0.22	3976	155.0	0.34
Milk, whole	Dairy	124	0.21	4574	161.3	0.34
Old cheese, 48+	Dairy	740	1.31	4359	27.0	0.35
Cheese spread, 48+	Dairy	467	0.88	3995	42.8	0.38
Green beans (plastic)	Vegetable	54	0.11	4860	370.4	0.39
Figs	Fruit	54	0.11	1446	370.4	0.40
Figs, dried	Fruit	162	0.34	1407	123.5	0.42
Yogurt drink, sweetener	Dairy	103	0.22	7725	194.2	0.43
Tofu	Meat/dairy substitutes	188	0.43	3743	106.4	0.46
Chicory	Vegetable	22	0.05	2605	909.1	0.46
Green beans frozen (plastic)	Vegetable	69	0.16	4566	289.9	0.47
Custard, vanilla	Dairy	86	0.20	2081	232.6	0.47
Chocolate milk, semi-skimmed	Dairy	102	0.24	2981	196.1	0.47
Kiwi	Fruit	30	0.07	1089	666.7	0.48
Chocolate milk, whole	Dairy	102	0.25	2579	196.1	0.49
Custard, whole, various flavors	Dairy	64	0.20	1516	312.5	0.64
Orange	Fruit	23	0.08	1078	869.6	0.68
Tangerine	Fruit	25	0.09	1223	800.0	0.69
Cottage cheese, whole	Dairy	125	0.47	2180	160.0	0.76
Green beans (can)	Vegetable	49	0.19	4240	408.2	0.78
Canned salmon, wild caught	Fish	91	0.37	1402	219.8	0.82
Green beans (glass)	Vegetable	49	0.20	4240	408.2	0.82
Cream/vanilla ice cream	Dairy	103	0.46	1130	194.2	0.90
Mozzarella	Dairy	160	0.85	1423	125.0	1.06
Cauliflower	Vegetable	25	0.14	2557	800.0	1.09
Broccoli	Vegetable	33	0.18	2750	606.1	1.11
Canned salmon, farmed fish	Fish	91	0.59	1402	219.8	1.29
Chicken egg	Eggs	64	0.43	1125	312.5	1.35
Garden peas with carrots (can)	Vegetable	29	0.20	1165	689.7	1.38
Plaice	Fish	101	0.73	1171	198.0	1.44
Garden peas with carrots (glass)	Vegetable	29	0.21	1165	689.7	1.44
Apricots	Fruit	20	0.15	1023	1000.0	1.47
Coffee	Non-alcoholic drinks	4	0.03	9000	5000.0	1.48
Gourmet	Fish	101	0.82	1077	198.0	1.62
Goat cheese, fresh	Dairy	101	0.85	1098	198.0	1.68
Cucumber with peel	Vegetable	20	0.19	3462	1000.0	1.88
Dutch shrimps	Fish	134	1.54	3207	149.3	2.30
Bean sprouts	Vegetable	16	0.20	1440	1250.0	2.48
Soda, sugar and caffeine	Non-alcoholic drinks	5	0.06	1023	4000.0	2.50
Zucchini	Vegetable	20	0.26	2368	1000.0	2.63
Strawberries	Fruit	15	0.64	1034	1333.3	8.55

^a^ Data from the Dutch food composition database (NEVO) [158]. ^b^ Data from the RIVM-database-milieubelasting-voedingsmiddelen [157]. ^c^ Calculated from ^a^ and ^b^.

## Data Availability

Data sharing not applicable.

## Supplementary Materials

The following are available online at https://www.mdpi.com/article/10.3390/nu14010180/s1, Table S1: Percentage of various nutrients supplied by the portion size determined to supply 20% of total required calcium (1000 mg/day) for Dutch adults.
