# Supplementary material for: Calcium Absorption from Food Products: Food Matrix Effects
Source: Nutrients. 2021 Dec 30;14(1):180. doi: 10.3390/nu14010180 (PMC8746734; doi:10.3390/nu14010180)
Supplement: Supplementary file 1 [file nutrients-14-00180-s001.zip › nutrients-1478158-supplementary.pdf]

nutrients

MDPI

**Table S1.** Percentage of various nutrients supplied by the portion size determined to supply 20% of total required calcium (1000 mg/day) for Dutch adult. Portion sizes are outlined in Table 9.

| Food Item | Food Category | Protein | LA | ALA | Fibre | Calcium | Iodine | Phos-phorus | Sele-nium | Iron | Copper | Magne-sium | Zinc | Cobala-min | Folate | Niacin | Ribofla-vin | Thiamin | Vitamin A | Vitamin B6 | Vitamin C | Vitamin D | Vitamin K |
|---|---|---|---|---|---|---|---|---|---|---|---|---|---|---|---|---|---|---|---|---|---|---|---|
| Seaweed kelp, raw | Miscellaneous | 3.5 | 0.2 | 0.0 | 6.2 | 20.0 | 5994 | 9.1 | 1.7 | 0.0 | 9.7 | 41.2 | 15.6 | 0.0 | 64.9 | 37.2 | 11.2 | 6.3 | 1.9 | 0.1 | 3.2 | 0.0 | 0.0 |
| Flax seed | Nuts and seeds | 25.6 | 31.1 | 1042 | 109.2 | 20.0 | 0.3 | 94.1 | 0.0 | 0.0 | 0.0 | 40.6 | 65.1 | 0.0 | 20.7 | 152.0 | 7.8 | 136.1 | 0.0 | 0.0 | 0.0 | 0.0 | 0.0 |
| Kale | Vegetable | 4.0 | 1.1 | 18.0 | 12.1 | 20.0 | 1.2 | 7.2 | 2.5 | 0.0 | 1.6 | 3.2 | 1.8 | 0.0 | 25.4 | 21.6 | 1.6 | 0.0 | 40.4 | 4.6 | 19.7 | 0.0 | 1011 |
| Vegetarian schnitzel | Meat/dairy substitutes | 5.3 | 10.7 | 0.2 | 5.0 | 20.0 | 0.0 | 26.4 | 1.8 | 0.0 | 0.0 | 1.4 | 4.0 | 3.0 | 0.9 | 0.0 | 2.7 | 2.9 | 0.0 | 6.6 | 0.0 | 0.0 | 0.0 |
| Cheese, 20+ | Dairy | 11.2 | 0.4 | 2.4 | 0.0 | 20.0 | 2.9 | 24.4 | 4.0 | 0.0 | 0.6 | 2.3 | 9.4 | 13.2 | 1.6 | 1.2 | 4.7 | 0.6 | 2.6 | 0.5 | 0.2 | 0.1 | 9.4 |
| Cheese Edam, 40+ | Dairy | 9.8 | 0.9 | 5.5 | 0.0 | 20.0 | 2.7 | 24.4 | 2.6 | 0.0 | 0.6 | 2.0 | 9.0 | 10.5 | 1.7 | 1.4 | 2.9 | 0.7 | 6.6 | 0.8 | 0.2 | 0.4 | 0.7 |
| Lettuce, medium | Vegetable | 9.1 | 1.9 | 18.1 | 19.6 | 20.0 | 4.8 | 24.0 | 0.0 | 0.0 | 9.4 | 16.2 | 13.2 | 0.0 | 28.0 | 70.8 | 11.8 | 20.0 | 65.9 | 13.3 | 3.4 | 0.0 | 400.0 |
| Herring in tomato sauce | Fish | 34.4 | 5.9 | 13.9 | 0.0 | 20.0 | 16.0 | 60.6 | 45.7 | 6.1 | 10.0 | 23.2 | 7.5 | 346.7 | 4.0 | 441.7 | 16.7 | 0.0 | 7.3 | 7.8 | 0.0 | 55.1 | 0.0 |
| Spinach, frozen | Vegetable | 4.9 | 0.5 | 20.7 | 11.9 | 20.0 | 1.6 | 9.9 | 0.0 | 0.0 | 6.9 | 12.0 | 10.9 | 0.0 | 23.6 | 84.9 | 8.5 | 7.8 | 105.5 | 3.6 | 9.0 | 0.0 | 1014 |
| Buttermilk | Dairy | 9.5 | 0.0 | 0.0 | 0.0 | 20.0 | 18.2 | 30.0 | 2.6 | 0.0 | 1.1 | 5.2 | 8.0 | 7.8 | 4.3 | 11.5 | 17.2 | 3.9 | 0.7 | 3.2 | 0.0 | 0.0 | 6.6 |
| Lettuce, crop | Vegetable | 9.8 | 3.3 | 35.9 | 19.6 | 20.0 | 6.8 | 26.7 | 0.0 | 0.0 | 10.2 | 17.5 | 17.8 | 0.0 | 35.1 | 76.5 | 15.3 | 21.6 | 75.6 | 10.8 | 0.0 | 0.0 | 752.2 |
| Yoghurt, low-fat | Dairy | 9.3 | 0.0 | 0.0 | 0.0 | 20.0 | 13.1 | 28.2 | 1.9 | 0.0 | 0.8 | 4.9 | 8.5 | 9.2 | 5.1 | 8.2 | 14.0 | 2.8 | 0.4 | 3.1 | 1.2 | 0.0 | 0.2 |
| Root | Vegetable | 8.9 | 11.1 | 5.9 | 85.9 | 20.0 | 12.3 | 39.1 | 0.0 | 0.0 | 18.5 | 14.8 | 17.3 | 0.0 | 62.4 | 277.8 | 13.9 | 23.5 | 1373 | 34.9 | 20.2 | 0.0 | 78.3 |
| Onion | Vegetable | 15.4 | 3.4 | 0.0 | 74.5 | 20.0 | 13.8 | 41.4 | 9.9 | 0.0 | 25.9 | 17.7 | 21.3 | 0.0 | 49.5 | 86.2 | 4.3 | 29.2 | 0.0 | 48.7 | 31.3 | 0.0 | 3.9 |
| Milk, skim | Dairy | 10.1 | 0.0 | 0.0 | 0.0 | 20.0 | 15.8 | 30.6 | 2.3 | 0.0 | 1.0 | 5.4 | 7.8 | 17.5 | 3.4 | 19.8 | 17.9 | 6.7 | 0.2 | 3.7 | 0.0 | 0.0 | 0.0 |
| Cheese Gouda,48+ | Dairy | 9.7 | 1.2 | 7.6 | 0.0 | 20.0 | 3.4 | 24.0 | 4.2 | 0.0 | 0.6 | 2.3 | 9.0 | 12.3 | 1.9 | 1.5 | 4.3 | 0.3 | 11.3 | 0.6 | 0.0 | 0.5 | 23.7 |
| Yoghurt, whole | Dairy | 9.1 | 0.6 | 1.1 | 0.0 | 20.0 | 13.9 | 29.0 | 4.0 | 0.0 | 0.9 | 5.2 | 7.9 | 8.4 | 5.3 | 17.5 | 14.0 | 3.0 | 6.0 | 2.5 | 0.0 | 0.0 | 2.6 |
| Milk, semi-skimmed | Dairy | 9.5 | 0.3 | 1.3 | 0.0 | 20.0 | 16.2 | 30.7 | 2.3 | 0.0 | 1.0 | 5.6 | 7.1 | 18.3 | 3.2 | 10.2 | 18.3 | 6.9 | 3.5 | 2.9 | 1.5 | 0.0 | 1.6 |
| Yoghurt, semi-skimmed | Dairy | 10.4 | 0.3 | 1.2 | 0.0 | 20.0 | 14.3 | 23.0 | 2.1 | 0.0 | 0.9 | 4.9 | 6.9 | 14.0 | 3.9 | 9.0 | 18.0 | 4.6 | 3.3 | 2.5 | 2.6 | 0.0 | 1.4 |
| Yoghurt, low-fat with fruits | Dairy | 9.1 | 0.0 | 0.0 | 0.6 | 20.0 | 13.1 | 28.5 | 2.2 | 0.0 | 1.9 | 5.3 | 8.6 | 8.9 | 5.3 | 19.4 | 13.6 | 3.3 | 0.6 | 2.7 | 1.4 | 0.0 | 0.4 |
| Milk, whole | Dairy | 9.2 | 0.8 | 2.6 | 0.0 | 20.0 | 16.0 | 30.5 | 4.6 | 0.0 | 1.0 | 5.5 | 7.9 | 16.1 | 3.2 | 10.1 | 18.1 | 5.1 | 8.0 | 2.8 | 0.0 | 0.0 | 3.2 |
| Almonds, peeled | Nuts and seeds | 26.4 | 101.8 | 0.0 | 19.8 | 20.0 | 1.2 | 80.3 | 2.0 | 0.0 | 28.7 | 61.6 | 23.3 | 0.0 | 5.6 | 189.9 | 30.9 | 11.2 | 0.1 | 2.5 | 0.0 | 0.0 | 0.0 |
| Old cheese, 48+ | Dairy | 11.3 | 1.4 | 8.9 | 0.0 | 20.0 | 3.1 | 25.6 | 0.0 | 0.0 | 1.7 | 2.6 | 10.6 | 12.1 | 2.2 | 1.7 | 3.4 | 0.9 | 12.4 | 1.0 | 0.2 | 0.5 | 26.1 |
| Cheese spread, 48+ | Dairy | 9.8 | 1.6 | 7.9 | 0.9 | 20.0 | 2.6 | 60.8 | 4.9 | 0.0 | 0.8 | 2.6 | 9.1 | 6.9 | 1.7 | 2.7 | 6.4 | 0.9 | 12.3 | 0.4 | 0.4 | 0.0 | 7.8 |
| Green beans (plastic) | Vegetable | 11.5 | 2.2 | 17.8 | 43.0 | 20.0 | 7.4 | 28.3 | 0.0 | 0.0 | 18.5 | 28.6 | 14.6 | 0.0 | 56.9 | 115.7 | 18.5 | 23.5 | 26.2 | 15.5 | 3.4 | 0.0 | 84.7 |
| figs | Fruit | 6.4 | 0.0 | 0.0 | 29.6 | 20.0 | 0.0 | 21.5 | 0.0 | 0.0 | 13.9 | 15.9 | 11.8 | 0.0 | 9.0 | 115.7 | 11.6 | 23.5 | 12.3 | 24.0 | 10.1 | 0.0 | 24.9 |

| | | | | | | | | | | | | | | | | | | | | | | | |
|---|---|---|---|---|---|---|---|---|---|---|---|---|---|---|---|---|---|---|---|---|---|---|---|
| Figs, dried | Fruit | 7.0 | 4.3 | 0.0 | 48.4 | 20.0 | 0.0 | 15.0 | 1.8 | 0.0 | 22.4 | 24.0 | 7.2 | 0.0 | 3.4 | 46.3 | 6.2 | 11.8 | 1.8 | 8.0 | 1.1 | 0.0 | 27.5 |
| Yoghurt drink, sweetener | Dairy | 10.4 | 0.0 | 0.0 | 0.0 | 20.0 | 16.2 | 27.2 | 2.8 | 0.0 | 1.2 | 5.5 | 6.2 | 12.6 | 4.7 | 12.1 | 15.8 | 6.2 | 0.3 | 3.4 | 1.8 | 0.0 | 0.3 |
| Tofu | Meat/dairy substitutes | 21.2 | 36.6 | 39.1 | 1.3 | 20.0 | 0.0 | 25.1 | 0.0 | 0.0 | 0.0 | 21.3 | 12.4 | 0.0 | 4.8 | 6.6 | 1.3 | 7.9 | 0.0 | 0.0 | 0.0 | 0.0 | 0.0 |
| Chicory | vegetable | 20.3 | 6.4 | 7.3 | 43.6 | 20.0 | 2.4 | 56.2 | 0.0 | 0.0 | 51.1 | 26.0 | 19.3 | 0.0 | 27.5 | 113.6 | 17.0 | 48.1 | 0.0 | 19.8 | 0.0 | 0.0 | 0.0 |
| Green beans frozen (plastic) | Vegetable | 9.0 | 0.6 | 4.6 | 24.3 | 20.0 | 5.8 | 19.5 | 0.0 | 0.0 | 10.9 | 16.6 | 7.7 | 0.0 | 14.9 | 72.5 | 9.1 | 15.3 | 24.7 | 2.0 | 13.2 | 0.0 | 99.4 |
| Custard, vanilla | Dairy | 8.8 | 0.9 | 1.9 | 1.9 | 20.0 | 18.4 | 30.4 | 3.3 | 0.0 | 1.5 | 6.0 | 7.2 | 14.5 | 1.4 | 14.5 | 17.4 | 4.9 | 9.0 | 4.1 | 0.0 | 0.0 | 4.0 |
| Chocolate milk, semi-skimmed | Dairy | 11.1 | 0.6 | 1.6 | 3.9 | 20.0 | 17.5 | 33.5 | 2.8 | 0.0 | 7.4 | 10.1 | 10.2 | 6.9 | 2.4 | 24.5 | 18.4 | 6.2 | 3.9 | 1.2 | 0.0 | 1.3 | 0.6 |
| Kiwi | Fruit | 10.3 | 10.7 | 64.0 | 61.3 | 20.0 | 0.0 | 41.2 | 9.5 | 0.0 | 50.0 | 26.7 | 14.9 | 0.0 | 69.1 | 125.0 | 8.3 | 7.1 | 6.2 | 28.2 | 478.8 | 0.0 | 104.8 |
| Chocolate milk, whole | Dairy | 11.8 | 1.2 | 1.6 | 3.9 | 20.0 | 17.5 | 31.0 | 2.8 | 0.0 | 3.7 | 10.6 | 9.6 | 16.2 | 2.4 | 24.5 | 18.4 | 4.1 | 8.4 | 3.5 | 3.6 | 0.0 | 0.8 |
| Custard, whole, various flavors | Dairy | 11.3 | 1.3 | 2.5 | 1.3 | 20.0 | 24.8 | 25.6 | 0.0 | 0.0 | 5.9 | 5.4 | 9.0 | 14.8 | 1.9 | 19.5 | 19.5 | 6.6 | 10.8 | 3.7 | 2.8 | 0.0 | 5.4 |
| Orange | Fruit | 12.0 | 24.3 | 76.5 | 69.6 | 20.0 | 14.5 | 37.9 | 0.0 | 0.0 | 21.7 | 24.8 | 15.7 | 0.0 | 85.9 | 217.4 | 16.3 | 55.2 | 17.4 | 30.7 | 403.2 | 0.0 | 1.2 |
| Tangerine | Fruit | 9.6 | 3.2 | 12.8 | 28.8 | 20.0 | 0.0 | 27.6 | 0.0 | 0.0 | 15.0 | 22.9 | 6.8 | 0.0 | 54.8 | 150.0 | 15.0 | 59.3 | 42.7 | 35.8 | 290.9 | 0.0 | 1.1 |
| Cottage cheese, whole | Dairy | 19.3 | 1.9 | 5.1 | 0.0 | 20.0 | 15.9 | 43.6 | 0.0 | 0.0 | 2.0 | 5.5 | 9.0 | 28.0 | 5.3 | 10.0 | 25.0 | 10.2 | 26.7 | 3.8 | 1.5 | 0.0 | 58.3 |
| Green beans (can) | Vegetable | 11.2 | 2.0 | 16.3 | 42.4 | 20.0 | 8.2 | 14.8 | 0.0 | 0.0 | 20.4 | 14.0 | 11.3 | 0.0 | 33.4 | 102.0 | 12.8 | 13.0 | 26.7 | 7.2 | 11.1 | 0.0 | 93.3 |
| Canned salmon, wild caught | Fish | 77.2 | 2.4 | 12.3 | 0.0 | 20.0 | 86.4 | 67.9 | 78.5 | 4.0 | 6.9 | 15.7 | 18.7 | 219.8 | 8.0 | 810.4 | 30.2 | 4.7 | 9.1 | 27.1 | 0.0 | 159.7 | 0.0 |
| Green beans (glass) | Vegetable | 11.2 | 2.0 | 16.3 | 42.4 | 20.0 | 8.2 | 14.8 | 0.0 | 0.0 | 20.4 | 14.0 | 11.3 | 0.0 | 33.4 | 102.0 | 12.8 | 13.0 | 26.7 | 7.2 | 11.1 | 0.0 | 93.3 |
| Cream/vanilla ice cream | Dairy | 10.4 | 3.5 | 9.3 | 2.3 | 20.0 | 12.6 | 30.7 | 5.5 | 0.0 | 1.2 | 7.2 | 4.1 | 19.9 | 1.1 | 12.1 | 26.7 | 8.2 | 48.2 | 4.2 | 0.0 | 1.3 | 8.9 |
| Mozzarella | Dairy | 40.2 | 3.3 | 9.0 | 0.0 | 20.0 | 15.0 | 79.5 | 5.4 | 0.0 | 0.0 | 3.6 | 34.6 | 53.1 | 7.6 | 31.3 | 21.1 | 4.0 | 47.2 | 7.4 | 0.0 | 1.7 | 20.7 |
| Cauliflower | Vegetable | 24.8 | 2.4 | 108.8 | 73.6 | 20.0 | 13.3 | 68.4 | 0.0 | 0.0 | 25.0 | 20.6 | 22.1 | 0.0 | 230.5 | 250.0 | 25.0 | 16.9 | 0.0 | 29.6 | 160.0 | 0.0 | 325.7 |
| Broccoli | Vegetable | 40.7 | 1.2 | 67.9 | 65.5 | 20.0 | 8.1 | 106.9 | 0.0 | 0.0 | 22.7 | 32.9 | 40.0 | 0.0 | 126.2 | 189.4 | 26.5 | 32.1 | 93.7 | 27.5 | 209.4 | 0.0 | 1351 |
| Canned salmon, farmed fish | Fish | 77.2 | 2.4 | 12.3 | 0.0 | 20.0 | 86.4 | 67.9 | 78.5 | 4.0 | 6.9 | 15.7 | 18.7 | 219.8 | 8.0 | 810.4 | 30.2 | 4.7 | 9.1 | 27.1 | 0.0 | 159.7 | 0.0 |
| Chicken egg | Eggs | 66.2 | 17.5 | 2.5 | 0.0 | 20.0 | 74.0 | 140.3 | 89.3 | 0.0 | 13.7 | 13.4 | 54.2 | 120.3 | 56.2 | 0.0 | 68.4 | 26.5 | 89.6 | 20.2 | 0.0 | 37.5 | 1.3 |
| Garden peas with carrots (can) | Vegetable | 40.4 | 10.3 | 60.7 | 132.4 | 20.0 | 9.2 | 72.7 | 9.9 | 0.0 | 43.1 | 25.6 | 42.6 | 0.0 | 46.0 | 215.5 | 21.6 | 43.8 | 292.4 | 21.5 | 6.3 | 0.0 | 129.1 |
| Plaice | Fish | 75.0 | 0.0 | 0.0 | 0.0 | 20.0 | 40.9 | 45.0 | 135.8 | 3.6 | 3.7 | 17.0 | 21.1 | 99.0 | 16.2 | 581.7 | 13.6 | 46.1 | 2.1 | 9.1 | 3.6 | 14.5 | 0.0 |
| Garden peas with carrots (glass) | Vegetable | 40.4 | 10.3 | 60.7 | 132.4 | 20.0 | 9.2 | 72.7 | 9.9 | 0.0 | 43.1 | 25.6 | 42.6 | 0.0 | 46.0 | 215.5 | 21.6 | 43.8 | 292.4 | 21.5 | 6.3 | 0.0 | 129.1 |
| Apricots | Fruit | 15.5 | 0.0 | 0.0 | 68.0 | 20.0 | 0.0 | 36.4 | 0.0 | 0.0 | 37.5 | 31.4 | 10.6 | 0.0 | 15.2 | 500.0 | 31.3 | 63.5 | 278.7 | 35.3 | 45.5 | 0.0 | 47.1 |
| Coffee | Non-alcoholic drinks | 17.2 | 0.0 | 0.0 | 0.0 | 20.0 | 13.3 | 0.0 | 0.0 | 0.0 | 31.3 | 85.7 | 5.3 | 0.0 | 0.0 | 1250 | 31.3 | 52.9 | 0.0 | 0.0 | 0.0 | 0.0 | 0.0 |
| Gourmet | Fish | 76.3 | 72.5 | 65.0 | 1.6 | 20.0 | 130.7 | 45.0 | 135.8 | 1.8 | 3.7 | 17.0 | 12.6 | 44.6 | 2.4 | 581.7 | 13.6 | 8.4 | 2.6 | 9.1 | 0.0 | 18.5 | 0.0 |
| Goat cheese, fresh | Dairy | 45.7 | 8.9 | 20.6 | 0.0 | 20.0 | 6.6 | 82.4 | 0.0 | 0.0 | 0.0 | 6.2 | 10.5 | 14.4 | 13.2 | 0.0 | 37.1 | 2.1 | 35.4 | 0.0 | 0.0 | 1.3 | 5.1 |
| Cucumber with peel | Vegetable | 12.0 | 9.0 | 64.0 | 24.0 | 20.0 | 20.0 | 58.2 | 0.0 | 0.0 | 18.8 | 31.4 | 67.0 | 0.0 | 18.8 | 125.0 | 12.5 | 10.6 | 20.0 | 20.0 | 36.4 | 0.0 | 298.6 |
| Dutch shrimps | Fish | 50.9 | 0.4 | 1.2 | 0.0 | 20.0 | 54.7 | 37.7 | 66.1 | 1.4 | 66.2 | 14.9 | 28.6 | 626.1 | 5.7 | 121.3 | 31.7 | 4.7 | 0.2 | 3.7 | 0.0 | 1.0 | 0.0 |
| Bean sprouts | Vegetable | 47.3 | 5.0 | 10.0 | 60.0 | 20.0 | 0.0 | 84.1 | 17.9 | 0.0 | 78.1 | 53.6 | 39.9 | 0.0 | 116.7 | 781.3 | 54.7 | 79.4 | 1.7 | 14.0 | 90.9 | 0.0 | 48.2 |

| | | | | | | | | | | | | | | | | | | | | | | | |
|---|---|---|---|---|---|---|---|---|---|---|---|---|---|---|---|---|---|---|---|---|---|---|---|
| Soda, sugar and caffeine | Non-alcoholic drinks | 0.0 | 0.0 | 0.0 | 0.0 | 20.0 | 0.0 | 138.2 | 0.0 | 0.0 | 25.0 | 0.0 | 0.0 | 0.0 | 0.0 | 0.0 | 0.0 | 0.0 | 0.0 | 0.0 | 0.0 | 0.0 | 0.0 |
| Zucchini | Vegetable | 25.8 | 1.0 | 32.0 | 44.0 | 20.0 | 14.7 | 92.7 | 14.3 | 0.0 | 6.3 | 62.9 | 33.0 | 0.0 | 93.9 | 312.5 | 31.3 | 52.9 | 44.0 | 55.9 | 27.3 | 0.0 | 60.0 |
| Strawberries | Fruit | 16.1 | 0.0 | 0.0 | 58.7 | 20.0 | 0.0 | 70.3 | 0.0 | 0.0 | 33.3 | 49.5 | 17.0 | 0.0 | 262.6 | 250.0 | 25.0 | 28.2 | 3.6 | 43.9 | 727.3 | 0.0 | 57.1 |
